# Sex-Specific Associations of Anxiety With Left Ventricular Hypertrophy and Transmural Dispersion of Repolarization in Hypertensive Patients

**DOI:** 10.3389/fcvm.2022.858097

**Published:** 2022-06-09

**Authors:** Ling Zhu, Qianwei Cui, Yong Zhang, Fuqiang Liu, Jingsha Zhao, Junkui Wang

**Affiliations:** ^1^Department of Cardiology, Shaanxi Provincial People’s Hospital, Xi’an, China; ^2^Department of Cardiology, The Third Affiliated Hospital of Xi’an Jiaotong University, Xi’an, China; ^3^Department of Intensive Care Unit, Third People’s Hospital of Chengdu, Chengdu, China

**Keywords:** anxiety, hypertension, left ventricular hypertrophy, sex, transmural dispersion of repolarization

## Abstract

**Background:**

The previous studies have shown that individuals with hypertension and anxiety have a higher mean left ventricular mass index (LVMI) and QTc dispersion. We explored the associations between anxiety and left ventricular hypertrophy (LVH) and between anxiety and transmural dispersion of repolarization (TDR) (as detected by T peak-T end interval/QT interval, Tp–Te/QT ratio) in patients with hypertension.

**Methods:**

A total of 353 patients with uncomplicated hypertension from the Shaanxi Provincial People’s Hospital were enrolled between 2017 and 2021. Anxiety was defined as a Hamilton Anxiety Scale (HAM-A) score ≥ 14. Logistic regression models were used to estimate the association between HAM-A and LVH. The association between HAM-A score and Tp–Te/QT was estimated using linear regression.

**Results:**

Participants were divided into two groups based on the presence of anxiety. LVMI was significantly higher in patients with hypertension and anxiety than in those with hypertension without anxiety (no anxiety: 84.36 ± 23.82, anxiety: 105.75 ± 25.45 g/m^2^, *p* < 0.001). HAM-A score was positively correlated with LVMI (*r* = 0.578, *p* < 0.001) and with Tp–Te/QT (*r* = 0.252, *p* < 0.001). Logistic regression models showed that patients with hypertension and anxiety were at higher risk of LVH than were patients with hypertension without anxiety (adjusted *OR*, 2.44, 95% *CI*, 1.35–4.43, *p* = 0.003). The linear regression analysis showed that the HAM-A score was associated with Tp–Te/QT ratio (adjusted β, 0.001, 95% *CI*, 0.001–0.002, *p* = 0.013). There was an interaction between sex and anxiety for LVH risk (*p* for interaction = 0.035) and for increased Tp–Te/QT (*p* for interaction = 0.014). After stratification by sex, anxiety was associated with increased risk for LVH in men with hypertension (adjusted *OR*, 5.56, 95% *CI*, 2.07–14.98, *p* = 0.001), but not in women (adjusted: *OR*, 1.44, 95% *CI*, 0.64–3.26, *p* = 0.377) with hypertension. The HAM-A score was also positively associated with Tp–Te/QT ratio in male (adjusted β, 0.002, 95% *CI*, 0.001–0.003, *p* < 0.001), but not in women (adjusted β, 0.001, 95% *CI*, –0.0002–0.002, *p* = 0.165).

**Conclusion:**

Our results indicated that anxiety was associated with LVH and with increased TDR in men with hypertension, but not in women with hypertension.

## Introduction

Left ventricular hypertrophy (LVH), which results from hypertension, is a strong predictor of future risk of ventricular arrhythmia and sudden cardiac death (1, 2). Increased dispersion of repolarization is a potential mechanism of ventricular arrhythmia and sudden cardiac death in hypertensive patients with LVH (3, 4). Reducing the heterogeneity of ventricular repolarization may mitigate arrhythmias and reduce the risk of sudden cardiac death in hypertensive patients with LVH (5–7). However, the heterogeneity in the incidence of LVH in patients with hypertension has rendered LVH unpredictable. Therefore, it is important to identify potential risk factors for LVH to reduce the burden of hypertension complications.

Anxiety is a common mood disorder. The previous studies showed that the anxiety was associated with cardiovascular diseases (8, 9). Hypertension with anxiety has been associated with higher mean left ventricular mass index (LVMI) and QTc dispersion (10). These findings provided evidence that anxiety may be closely associated with LVH and with a dispersion of repolarization in patients with hypertension. However, the impact of anxiety on LVH and transmural dispersion of repolarization (TDR) is not clear. In this study, we investigated the associations between anxiety and LVH and TDR [as determined by T peak-T end interval/QT interval, Tp–Te/QT ratio (11)] in patients with hypertension.

## Materials and Methods

### Study Subjects

A total of 353 patients with hypertension without complications were consecutively enrolled at Shaanxi Provincial People’s Hospital from November 2017 to May 2021. Patients with heart failure, coronary heart disease, atrial fibrillation, cardiomyopathy, diabetes, kidney disease, thyroid dysfunction, cancer, active infections, chronic immune-mediated disorders, or current use of immunosuppressive drugs including corticosteroids were excluded. Blood pressure was measured by experienced doctors. Blood pressure was recorded using two separate measurements taken at least 30 s apart after 5 min of rest. Hypertension was defined as a systolic blood pressure above 140 mmHg, a diastolic blood pressure above 90 mmHg, or use of anti-hypertensive medication within 2 weeks (12). Left ventricular mass (LVM) was determined as per the American Society of Echocardiography recommendations: 0.8 × {1.04 [(left ventricular internal diameter in diastole + left ventricular posterior wall thickness in diastole + left ventricular septal wall thickness in diastole)^3^- (left ventricular internal diameter in diastole)^3^]} + 0.6 g (13). Left ventricular hypertrophy was assessed using LVMI (indexed to body surface area, BSA, g/m^2^), which was defined as an LVMI greater than 115 (male) or 95 (female) (14). The study was approved by the Ethics Committee of Shaanxi Provincial People’s Hospital and was performed in accordance with the requirements of the Declaration of Helsinki. All patients gave informed consent to participate in this study.

### Clinical Data Collection

All subjects completed a full clinical assessment such as age, gender, body mass index (BMI), detailed medical history, clinical examination, 12-lead electrocardiogram [ECG (CardiMax FX-7542; Fukuda Denshi., Ltd., Tokyo, Japan)], and echocardiogram (EPIQ 7C, Philips, Netherlands). Baseline characteristics of patients with hypertension were collected by experienced physicians. The data were entered into the web database using the double entry method by different individuals (Likang Times Technology Ltd., Beijing, China). When two values were consistent, the data were entered into the database. Otherwise, the system flagged the discrepancy and corrected the error by checking the source data.

### Anxiety Assessment

All enrolled subjects completed the Hamilton Anxiety Scale (HAM-A), which quantifies the severity of anxiety based on symptoms in the preceding week. The scale consists of 14 items ranging from 0 to 4. The total score on the basis of HAM-A ranges from 0 to 56. Anxiety was defined as a HAM-A score ≥ 14. HAM-A scores were obtained from all subjects at enrollment.

### Blood Sampling

Venous blood was collected from all subjects after overnight fasting (>12 h) at enrollment. Serum biochemical variables (such as total cholesterol, low-density lipoprotein cholesterol, high-density lipoprotein cholesterol, triglycerides, uric acid, and creatinine) were measured by enzymatic methods using an automated analyzer (AU5800, Beckman Coulter, Brea, CA, United States).

### Measurement of T-Wave Peak to T-Wave End Interval

A speed of 25 mm/s and a voltage of 10.0 mm/mv were used for 12-lead ECG. The Tp–Te interval and QT interval were measured by a physician using the average of three consecutive cardiac cycles of the V6 lead. If lead V6 was not appropriately measured, it was replaced with leads V4 and V5. The QT interval was defined as the time from the onset of the QRS complex to the end of the T wave. The Tp–Te interval was measured from the peak of the T wave to the end of the T wave (15, 16). The peak of the T wave was defined as the highest point of the T wave. The endpoint of the T wave was defined as: (1) When the T wave pattern was normal, the T wave endpoint was identified as the point where the tangent on the descending limb of the T wave intersected with the baseline; (2) If the T wave was followed by the U wave, the lowest point between the T wave and the U wave was considered the T wave endpoint; (3) When the T-wave was biphasic, the T wave endpoint was measured at the nadir of the two waves and at the final return to baseline. The Tp–Te interval serves as an index of TDR (11). The Tp–Te/QT ratio eliminates the confounding effect of heart rate variability and can serve as a more sensitive index of arrhythmogenesis (17). The Tp–Te/QT ratio was calculated as the ratio of Tp–Te to the corresponding QT interval in that lead.

### Statistical Analysis

Data are expressed as mean ± *SD*. Continuous variables were compared using *t*-tests for parametric variables and the Mann–Whitney *U* test for non-parametric variables. The chi-square test was used to analyze categorical variables. The Spearman’s correlation test was used to analyze the correlation between HAM-A and LVMI or Tp–Te/QT. We conducted univariate and multivariate logistic regression analysis twice using HAM-A as continuous variables and categorical variables (HAM-A score ≥ 14) to investigate the association between anxiety and LVH in hypertensive patients. The association between HAM-A score and LVMI or Tp–Te/QT was estimated with univariate and multivariate linear regression model. Model 1 was unadjusted. Model 2 was adjusted for age, sex, BMI, and smoking. Model 3 was adjusted for age, sex, BMI, smoking, creatinine, uric acid, triglyceride, total cholesterol, low-density lipoprotein cholesterol, and high-density lipoprotein cholesterol, angiotensin converting enzyme inhibitor (ACEI)/angiotensin II receptor blocker (ARB), β blockers, and calcium channel blockers (CCB). Furthermore, we also performed subgroup analyses stratified by sex, age, BMI, and smoking. Interaction analyses were used to examine whether the association between anxiety and LVH differed based on sex, age (<60 years vs. ≥60 years), BMI (<24 kg/m^2^ vs. ≥24 kg/m^2^), or smoking. The *p*-values < 0.05 were considered statistically significant.

The PASS statistical software (version 15.0.1) was used to calculate the sample size. We used a sample size large enough to detect an odds ratio of 1.5 with 85% power at the 0.05 significance level with a two-sided test. The sample size calculated using the PASS software was greater than 350 cases based on the 19.3% prevalence of LVH in Chinese adults with hypertension (18).

A *p* value < 0.05 was considered statistically significant. All analyses were performed using the PASW Statistics 26.0 software (SPSS Inc., Chicago, IL, United States). All statistical tests were two-sided.

## Results

### Baseline Characteristics

Baseline characteristics of the 353 patients included in this study based on LVH are summarized in Table 1. Participants ranged from 28 to 84 years old (mean age: 59.29 ± 9.87 years), and 50.1% were male. HAM-A scores ranged from 0 to 38, and the mean HAM-A score for the entire study population was 10.99 ± 6.21. Subjects in the LVH group had higher systolic blood pressure (no-LVH: 128.49 ± 15.54, LVH: 142.70 ± 22.45 mmHg, *p* < 0.001), higher diastolic blood pressure (no-LVH: 78.08 ± 10.34, LVH: 84.52 ± 13.80 mmHg, *p* < 0.001), higher interventricular septum (no-LVH: 9.95 ± 0.90, LVH: 10.45 ± 1.15 mm, *p* < 0.001), thicker left ventricular posterior wall (no-LVH: 9.80 ± 0.81, LVH: 10.16 ± 1.04 mm, *p* = 0.006), higher HAM-A score (no-LVH: 9.92 ± 5.29, LVH: 14.31 ± 7.58, *p* < 0.001), higher LVMI (no-LVH: 77.74 ± 15.68, LVH: 123.57 ± 18.83 g/m^2^, *p* < 0.001), and higher Tp–Te/QT ratio (no-LVH: 0.23 ± 0.04, LVH: 0.27 ± 0.05 ms, *p* < 0.001). Subjects in the LVH group had higher percentage of ACEI/ARB use (no-LVH: 25.1%, LVH: 54.7%, *p* < 0.001) and CCB use (no-LVH: 24.3%, LVH: 39.5%, *p* = 0.006). In addition, the baseline characteristics of the 353 hypertensive patients grouped based on anxiety are listed in Supplementary Table 1.

**TABLE 1:** Baseline clinical characteristics of the patients with hypertension.

Variables	Total, *n* = 353	LVH (–), *n* = 267	LVH (+), *n* = 86	*p* value
Age, year	59.29 ± 9.87	59.25 ± 9.87	59.43 ± 9.94	0.755
Male, *n* (%)	177 (50.1)	137 (51.3)	40 (46.5)	0.459
BMI, kg/m^2^	24.47 ± 3.11	24.37 ± 3.03	24.79 ± 3.33	0.522
Heart rate, bpm	73.80 ± 10.27	73.88 ± 9.91	73.55 ± 11.36	0.810
SBP, mmHg	131.95 ± 18.48	128.49 ± 15.54	142.70 ± 22.45	<0.001
DBP, mmHg	79.65 ± 11.59	78.08 ± 10.34	84.52 ± 13.80	<0.001
Cigarette smoking, *n* (%)	95 (26.9)	73 (27.3)	22 (25.6)	0.782
Uric acid, umol/L	314.17 ± 88.10	308.68 ± 80.18	331.20 ± 107.88	0.321
Creatinine, mmol/L	65.99 ± 15.76	65.62 ± 15.28	67.14 ± 17.20	0.645
TG, mmol/L	1.69 ± 1.01	1.60 ± 0.86	1.95 ± 1.36	0.054
TC, mmol/L	4.16 ± 1.07	4.14 ± 1.03	4.21 ± 1.19	0.836
HDL-C, mmol/L	1.15 ± 0.28	1.15 ± 0.29	1.14 ± 0.26	0.863
LDL-C, mmol/L	2.33 ± 0.81	2.32 ± 0.76	2.38 ± 0.95	0.854
IVS, mm	10.07 ± 0.99	9.95 ± 0.90	10.45 ± 1.15	<0.001
LVPW, mm	9.89 ± 0.89	9.80 ± 0.81	10.16 ± 1.04	0.005
LVEDD, mm	46.20 ± 4.97	45.91 ± 5.20	47.08 ± 4.07	0.118
LVEF, %	62.92 ± 3.80	62.93 ± 3.68	62.90 ± 4.18	0.706
Prior medication, *n* (%)				
ACEI/ARB	114 (32.3)	67 (25.1)	47 (54.7)	<0.001
CCB	99 (28.0)	65 (24.3)	34 (39.5)	0.006
β blockers	129 (36.5)	93 (34.8)	36 (41.9)	0.239
Diuretics	18 (5.1)	12 (4.5)	6 (7.0)	0.363
HAM-A score	10.99 ± 6.21	9.92 ± 5.29	14.31 ± 7.58	<0.001
LVMI, g/m^2^	88.90 ± 25.68	77.74 ± 15.68	123.57 ± 18.83	<0.001
Tp–Te/QT	0.24 ± 0.04	0.23 ± 0.04	0.27 ± 0.05	<0.001

*Continuous variables are presented as mean ± SD; categorical variables are presented as numbers (percentages).*

*ACEI, angiotensin converting enzyme inhibitor; ARB, angiotensin II receptor blocker; BMI, body mass index; CCB, calcium channel blockers; DBP, diastolic blood pressure; HAM-A, Hamilton anxiety scale; HDL-C, high-density lipoprotein cholesterol; IVS, interventricular septum; LAD, left atrial diameter; LDL-C, low-density lipoprotein cholesterol; LVEDD, left ventricular end-diastolic diameter; LVEF, left ventricular ejection fraction; LVH, left ventricular hypertrophy; LVMI, left ventricular mass index; LVPW, left ventricular posterior wall; QT interval, Q wave start to T wave end interval; SBP, systolic blood pressure; TC, total cholesterol; TG, triglyceride; Tp–Te interval, T-wave peak to T-wave end interval.*

### Association of Anxiety With Left Ventricular Hypertrophy

**FIGURE 1:**
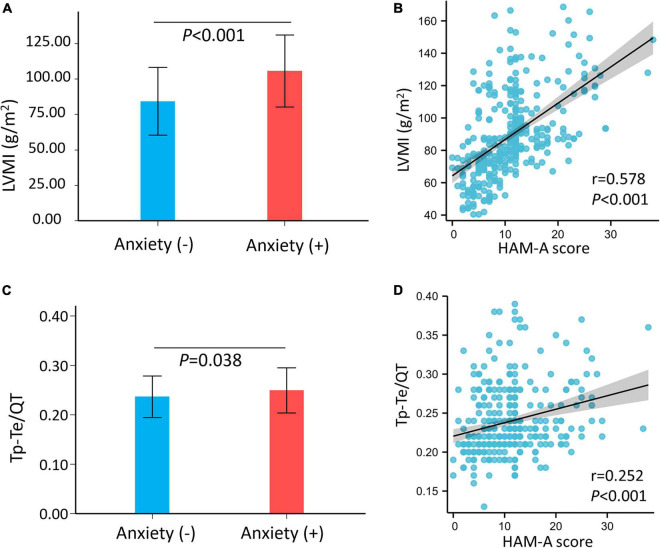
The left ventricular mass index (LVMI) and Tp–Te/QT ratio of hypertensive patients and correlation with Hamilton anxiety scale (HAM-A) score. **(A)** The LVMI was significantly higher in hypertensive patients with anxiety than hypertensive patients without anxiety (*p* < 0.001). **(B)** The HAM-A score was significantly positive correlated with LVMI (*r* = 0.578, *p* < 0.001). **(C)** The Tp–Te/QT ratio was significantly higher in hypertensive patients with anxiety than hypertensive patients without anxiety (*p* < 0.001). **(D)** HAM-A score was weakly positively correlated with Tp–Te/QT ratio in hypertensive patients (*r* = 0.252, *p* < 0.001). HAM-A, Hamilton anxiety scale; LVMI, left ventricular mass index; QT interval, Q wave start to T wave end interval; Tp–Te interval, T-wave peak to T-wave end interval.

The LVMI was markedly increased in patients with hypertension and anxiety compared with that in patients with hypertension without anxiety (no anxiety: 84.36 ± 23.82, anxiety: 105.75 ± 25.45 g/m^2^, *p* < 0.001) (Figure 1A). In addition, the HAM-A score was significantly positively correlated with LVMI (*r* = 0.578, *p* < 0.001) (Figure 1B). Univariate and multivariate linear regression analysis showed that HAM-A score was independently and positively associated with LVMI (Model 1: β, 2.24, 95% *CI*, 1.88–2.61, *p* < 0.001; Model 2: β, 2.33, 95% *CI*, 1.97–2.69, *p* < 0.001; Model 3: β, 2.13, 95% *CI*, 1.77–2.49, *p* < 0.001) (Table 2).

**TABLE 2:** Linear regression associations of HAM-A score with LVMI and Tp–Te/QT ratio.

Model 1*		Model 2^†^		Model 3^‡^	
	β (95% CI)	*P* value	β (95% CI)	*P* value	β (95% CI)	*P* value
LVMI	2.24 (1.88–2.61)	<0.001	2.33 (1.97–2.69)	<0.001	2.13 (1.77–2.49)	<0.001
Tp–Te/QT	0.002 (0.001–0.002)	<0.001	0.002 (0.001–0.003)	<0.001	0.001 (0.000–0.002)^§^	0.013

*Model 1: unadjusted.

*^†^Model 2: adjusted for age, gender, body mass index, cigarette smoking.*

*^‡^Model 3: adjusted for age, gender, body mass index, cigarette smoking, creatinine, uric acid, total cholesterol, triglyceride, low-density lipoprotein cholesterol, high-density lipoprotein cholesterol, ACEI/ARB, β blockers, and calcium channel blockers.*

*^§^Adjusted for Model 3 plus LVH.*

*ACEI, angiotensin converting enzyme inhibitor; ARB, angiotensin II receptor blocker; CI, confidence interval; HAM-A, Hamilton anxiety scale; LVH, left ventricular hypertrophy; LVMI, left ventricular mass index; OR, odds ratio; QT interval, Q wave start to T wave end interval; Tp–Te interval, T-wave peak to T-wave end interval.*

Univariate analysis using HAM-A as a continuous variable showed that every 1 point increase in HAM-A corresponded to a 12% increase in LVH risk (*OR*, 1.12, 95% *CI*, 1.07–1.16, *p* < 0.001). Multivariate analysis showed that anxiety (each additional 1 point) was independently and positively associated with hypertensive LVH (Model 2: *OR*, 1.12, 95% *CI*, 1.07–1.16, *p* < 0.001; Model 3: *OR*, 1.11, 95% *CI*, 1.06–1.16, *p* < 0.001) (Table 3).

**TABLE 3:** Logistic regression associations of HAM-A score as continuous and categorical variables with LVH.

HAM-A score	Model 1*		Model 2^†^		Model 3^‡^	
	OR (95% CI)	*P* value	OR (95% CI)	*P* value	OR (95% CI)	*P* value
Each additional 1 score of HAM-A	1.12 (1.07–1.16)	<0.001	1.12 (1.07–1.16)	<0.001	1.11 (1.06–1.16)	<0.001
HAM-A score ≥ 14^§^	2.86 (1.65–4.93)	<0.001	2.78 (1.59–4.83)	<0.001	2.44 (1.35–4.43)	0.003

*Model 1: unadjusted.

*^†^Model 2: adjusted for age, gender, body mass index, cigarette smoking.*

*^‡^Model 3: adjusted for age, gender, body mass index, cigarette smoking, creatinine, uric acid, total cholesterol, triglyceride, low-density lipoprotein cholesterol, high-density lipoprotein cholesterol, ACEI/ARB, β blockers, and calcium channel blockers.*

*^§^Compared with HAM-A score < 14.*

*ACEI, angiotensin converting enzyme inhibitor; ARB, angiotensin II receptor blocker; OR, odds ratio; CI, confidence interval; HAM-A, Hamilton anxiety scale; LVH, left ventricular hypertrophy.*

According to the definition of anxiety (HAM-A score ≥ 14), univariable logistic regression models (Model 1) using HAM-A score as a categorical variable showed that patients with hypertension and anxiety had a higher risk of LVH than patients with hypertension without anxiety (*OR*, 2.86, 95% *CI*, 1.65–4.93, *p* < 0.001). Compared with patients with hypertension without anxiety, the *OR* values for LVH in patients with hypertension and anxiety were 2.78 (Model 2: *OR*, 2.78, 95% *CI*, 1.59–4.83, *p* < 0.001) and 2.44 (Model 3: *OR*, 2.44, 95% *CI*, 1.35–4.43, *p* = 0.003) (Table 3).

### Association Between Hamilton Anxiety Scale and Transmural Dispersion of Repolarization

We also evaluated the association between HAM-A score and Tp–Te/QT ratio in patients with hypertension. Compared with patients with hypertension without anxiety, the Tp–Te/QT ratio (no anxiety: 0.24 ± 0.04, anxiety: 0.25 ± 0.05, *p* = 0.038) (Figure 1C) was higher in patients with hypertension and anxiety. In addition, HAM-A score was weakly positively correlated with Tp–Te/QT ratio (*r* = 0.252, *p* < 0.001) (Figure 1D) in patients with hypertension. Further analyses showed a significant correlation between LVMI and Tp–Te/QT ratio (*r* = 0.545, *p* < 0.001) (Supplementary Figure 1A).

Linear regression analysis showed that HAM-A score was associated with Tp–Te/QT ratio (β, 0.002, 95% *CI*, 0.001–0.002, *p* < 0.001). After adjusting for variables according to Model 2, HAM-A score was positively correlated with Tp–Te/QT ratio (β, 0.002, 95% *CI*, 0.001–0.003, *p* < 0.001). Adjustment for variables in Model 3 produced similar results to that obtained in Model 2 (β, 0.001, 95% *CI*, 0.001–0.002, *p* = 0.013) (Table 2).

### Subgroup Analysis of the Association Between Anxiety and Left Ventricular Hypertrophy

The association between anxiety and LVH significantly interacted with sex (*p* for interaction = 0.035) but not with age (<60 years vs. ≥60 years) (*p* for interaction = 0.380), BMI (<24 kg/m^2^ vs. ≥24 kg/m^2^) (*p* for interaction = 0.551), or smoking (*p* for interaction = 0.173).

**FIGURE 2:**
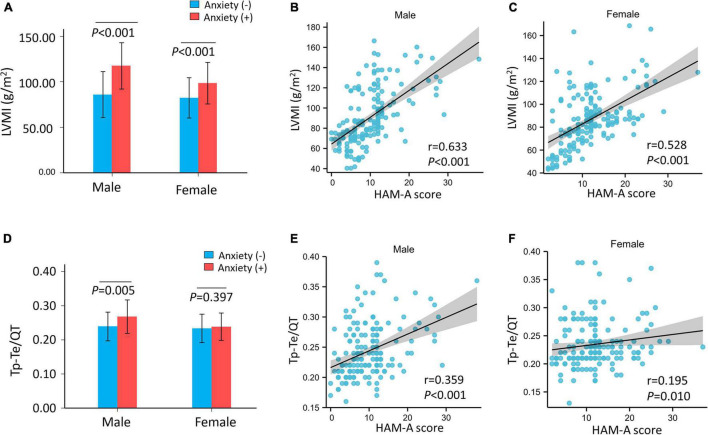
The LVMI and Tp–Te/QT ratio of hypertensive patients and correlation with HAM-A score after stratified by sex. **(A)** The LVMI was significantly higher in hypertensive patients with anxiety than hypertensive patients without anxiety both in male (*p* < 0.001) and female (*p* < 0.001). **(B,C)** HAM-A score was significantly positive correlated with LVMI both in male (*r* = 0.633, *p* < 0.001) and female (*r* = 0.528, *p* < 0.001). The Tp–Te/QT ratio was significantly higher in hypertensive patients with anxiety than hypertensive patients without anxiety (*p* < 0.001). **(D)** Tp–Te/QT ratio was higher in male (*p* = 0.005) with anxiety but not in female (*p* = 0.397) with anxiety in hypertensive patients. **(E,F)** HAM-A score was positively correlated with Tp–Te/QT ratio (male: *r* = 0.359, *p* < 0.001; female: *r* = 0.195, *p* = 0.010). HAM-A, Hamilton anxiety scale; LVMI, left ventricular mass index; QT interval, Q wave start to T wave end interval; Tp–Te interval, T-wave peak to T-wave end interval.

We performed stratified analyses by sex. The prevalence of anxiety in men and women was 15.8% (28/177) and 26.7% (47/176) (*p* = 0.012), respectively. Left ventricular mass index was higher in patients with hypertension and anxiety in men (no anxiety: 86.03 ± 25.19, anxiety: 117.62 ± 25.46 g/m^2^, *p* < 0.001) and in women with hypertension and anxiety (no anxiety: 82.43 ± 22.08, anxiety: 98.68 ± 22.90 g/m^2^, *p* < 0.001) than in men and women with hypertension without anxiety (Figure 2A). However, LVMI was elevated more significantly in men with anxiety than in women with anxiety. HAM-A score was significantly positive correlated with LVMI in men (*r* = 0.633, *p* < 0.001) (Figure 2B) and in women (*r* = 0.528, *p* < 0.001) (Figure 2C).

**FIGURE 3:**
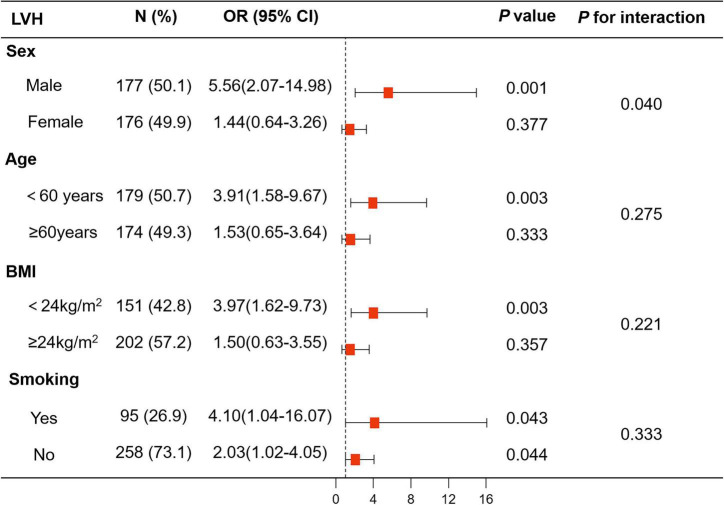
Logistic analysis of anxiety for LVH in subgroup analyses stratified by sex, age, body mass index (BMI), and smoking. The associations of anxiety with LVH showed interaction with sex, while there was no interaction with age (<60 years vs. ≥60 years), BMI (<24 kg/m^2^ vs. ≥24 kg/m^2^), and smoking. BMI, body mass index; LVH, left ventricular hypertrophy.

There was an interaction between sex and anxiety with regard to LVH risk (Model 1: *p* for interaction = 0.035, Model 2: *p* for interaction = 0.032, and Model 3: *p* for interaction = 0.040). In the logistic regression models, the risk of LVH was higher in the anxiety group than in the no anxiety group in male patients with hypertension (Model 1: *OR*, 5.73, 95% *CI*, 2.43–13.50, *p* < 0.001; Model 2: *OR*, 5.73, 95% *CI*, 2.43–13.56, *p* < 0.001; and Model 3: *OR*, 5.56, 95% *CI*, 2.07–14.98, *p* = 0.001). In contrast, there was no association between anxiety and LVH in female patients with hypertension (Model 1: *OR*, 1.70, 95% *CI*, 0.82–3.53, *p* = 0.152; Model 2: *OR*, 1.65, 95% *CI*, 0.79–3.43, *p* = 0.185; and Model 3: *OR*, 1.44, 95% *CI*, 0.64–3.26, *p* = 0.377) (Figure 3 and Supplementary Table 2).

The results of logistic regression analysis of anxiety for LVH in subgroups stratified by age, BMI, and smoking are shown in Figure 3 and Supplementary Table 2.

### Subgroup Analysis of the Association Between Hamilton Anxiety Scale and T-Wave Peak to T-Wave End/Q Wave Start to T Wave End Interval Ratio

The association between HAM-A score and Tp–Te/QT ratio showed a significant interaction with sex (*p* for interaction = 0.014), but not with age (<60 years vs. ≥60 years) (*p* for interaction = 0.595), BMI (<24 kg/m^2^ vs. ≥24 kg/m^2^) (*p* for interaction = 0.635), or smoking (*p* for interaction = 0.367).

After stratification by sex, the Tp–Te/QT ratio was higher in men with anxiety, but not in women with anxiety, than those without anxiety (male: no anxiety: 0.24 ± 0.04, anxiety: 0.27 ± 0.05 ms, *p* = 0.005; Tp–Te/QT ratio female: no anxiety: 0.23 ± 0.04, anxiety: 0.24 ± 0.04 ms, *p* = 0.397) (Figure 2D). The HAM-A score was positively correlated with Tp–Te/QT ratio (male: *r* = 0.359, *p* < 0.001; female: *r* = 0.195, *p* = 0.010) (Figures 2E,F) in men and in women. After stratification by sex, LVMI remained positively correlated with Tp–Te/QT ratio (male: *r* = 0.597, *p* < 0.001; female: *r* = 0.484, *p* < 0.001) (Supplementary Figures 1B,C).

**FIGURE 4:**
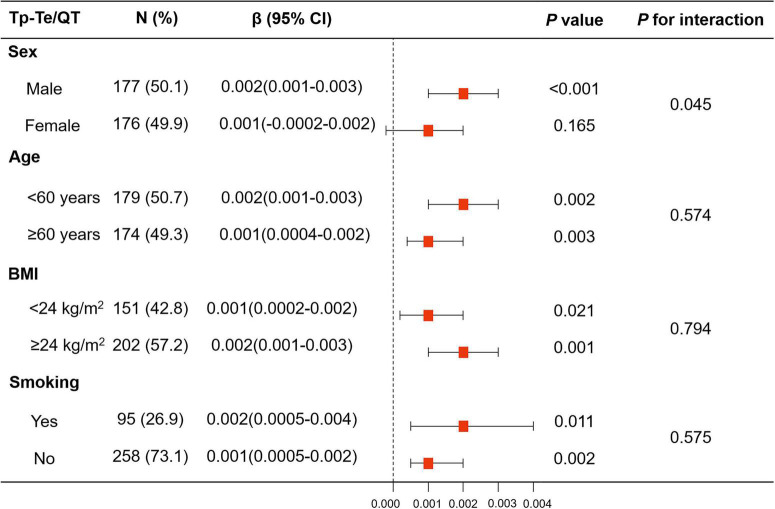
Linear regression model of HAM-A score for Tp–Te/QT ratio in subgroup analyses stratified by sex, age, BMI, and smoking. The associations of HAM-A score with Tp–Te/QT ratio showed significant interaction with sex, while there was no interaction with age (<60 years vs. ≥60 years), BMI (<24 kg/m^2^ vs. ≥24 kg/m^2^), and smoking. BMI, body mass index; HAM-A, Hamilton anxiety scale; QT interval, Q wave start to T wave end interval; Tp–Te interval, T-wave peak to T-wave end interval.

There was an interaction between anxiety and sex with regard to increased Tp–Te/QT ratio (Model 1: *p* for interaction = 0.014, Model 2: *p* for interaction = 0.017, and Model 3: *p* for interaction = 0.045). The linear regression analysis showed that the HAM-A score was associated with Tp–Te/QT ratio in men (Model 1: β, 0.003, 95% *CI*, 0.002–0.004, *p* < 0.001; Model 2: β, 0.003, 95% *CI*, 0.002–0.004, *p* < 0.001; and Model 3: β, 0.002, 95% *CI*, 0.001–0.003, *p* < 0.001), but not in women (Model 1: β, 0.001, 95% *CI*, –0.0001–0.002, *p* = 0.061; Model 2: β, 0.001, 95% *CI*, 0.0001–0.002, *p* = 0.050; and Model 3: β, 0.001, 95% *CI*, –0.0002–0.002, *p* = 0.165) (Figure 4 and Supplementary Table 3).

The results of linear regression analysis of anxiety and LVH in subgroups stratified by age, BMI, and smoking are shown in Figure 4 and Supplementary Table 3.

## Discussion

In this study, we found that anxiety was associated with LVH and with increased TDR in patients with hypertension, but an independent association was only found in men. These results showed that the associations between anxiety and LVH and between anxiety and increased TDR may predict the predisposition to cardiac arrhythmias in hypertensive patients.

Some studies have shown that anxiety is associated with hypertension (19, 20). Furthermore, the previous studies found that individuals with hypertension and anxiety had a higher mean LVMI (10, 21). However, these studies did not explore the potential link between anxiety and hypertensive LVH. Prior studies also showed that high anxiety was associated with increased QT dispersion in healthy participants (22). However, the effect of anxiety on TDR in patients with hypertension had not been evaluated. This study demonstrated an independent association between anxiety and LVH, and between anxiety and LVH-related increased Tp–Te/QT ratio in hypertensive patients. The associations between anxiety and hypertensive LVH and between anxiety and increased TDR associated with LVH were assessed using univariate and multivariate logistic regression models and linear regression models. To further assess the robustness of our findings, HAM-A scores were used as categorical and continuous variables. Several logistic regression models (Models 1–3) were used to examine the associations between anxiety and LVH and between anxiety and increased TDR. To eliminate the effects of other diseases, we enrolled patients with uncomplicated hypertension and excluded patients with NYHA III or IV, coronary heart disease, atrial fibrillation, cardiomyopathy, diabetes, kidney disease, thyroid dysfunction, cancer, active infections, chronic immune-mediated disorders, or current use of immunosuppressive drugs including corticosteroids. All analyses showed that anxiety was an independent risk factor for LVH and for increased TDR in hypertension.

The psychosocial stress caused by anxiety disorders increases circulating catecholamines through the hypothalamic–pituitary–adrenal axis. Chronic hyperstimulation of the hypothalamic centers may lead to activation of the sympathetic nervous system and the hypothalamic–pituitary–adrenal axis (23). Negative psychosocial factors have the potential to affect chronic sympathetic hyperactivity, which can lead to increased cardiovascular workload and increased risk of LVH and serious dysrhythmias (24, 25). Therefore, anxiety may promote LVH and increased TDR through chronic sympathetic hyperactivity in hypertensive patients.

We also performed subgroup analyses stratified by sex, age, BMI, and smoking. Interaction analyses found that the associations between anxiety and hypertensive LVH and between anxiety and TDR showed interactions with sex, but not with age, BMI, or smoking. Therefore, we performed subgroup analyses stratified by sex. The results showed that the prevalence of anxiety was significantly higher in men than that in women, which was consistent with the findings of previous studies (26). Our results showed that anxiety was associated with LVH and with increased Tp–Te/QT ratio in men, but not in women. A possible reason for this discrepancy is that the biological stress responses to anxiety differ between men and women. Men have higher salivary cortisol levels in response to psychological stress than women (27, 28). Men with anxiety may also experience greater perceived stress than women with anxiety. The findings of our study highlight the vulnerability of anxiety to the development of LVH in men with hypertension.

Our study had some limitations. This was a single-center study limited to individuals from the native Chinese population. In addition, HAM-A scores were only obtained at enrollment, and serial HAM-A assessments were not performed. Finally, the sample size in this study was small and only the sample size of the whole sample was calculated without considering the sample size of subgroups. These factors limited the generalizability of our findings.

## Conclusion

Our study showed that anxiety was associated with increased risk of LVH and with increased TDR, especially in men with hypertension. Mental health interventions to improve anxiety should be sensitive to sex differences, and should focus more heavily on men. Although a causal link between anxiety and LVH has not been established, our findings suggest that anxiety may be associated with the development of LVH and with the increased TDR in hypertensive patients.

## Data Availability Statement

The original contributions presented in the study are included in the article/Supplementary Material, further inquiries can be directed to the corresponding authors.

## Ethics Statement

The studies involving human participants were reviewed and approved by the Ethics Committee of Shaanxi Provincial People’s Hospital. The patients/participants provided their written informed consent to participate in this study.

## Author Contributions

LZ conducted research, performed data analysis, and wrote the manuscript. LZ and QC performed the data collection. YZ assisted in editing of the manuscript and provided statistical consultation. FL, JW, and JZ designed and planned the study and revised the manuscript. All authors reviewed and approved the manuscript.

## Conflict of Interest

The authors declare that the research was conducted in the absence of any commercial or financial relationships that could be construed as a potential conflict of interest.

## Publisher’s Note

All claims expressed in this article are solely those of the authors and do not necessarily represent those of their affiliated organizations, or those of the publisher, the editors and the reviewers. Any product that may be evaluated in this article, or claim that may be made by its manufacturer, is not guaranteed or endorsed by the publisher.
