# Supplementary material for: Sex-Specific Associations of Anxiety With Left Ventricular Hypertrophy and Transmural Dispersion of Repolarization in Hypertensive Patients
Source: Front Cardiovasc Med. 2022 Jun 9;9:858097. doi: 10.3389/fcvm.2022.858097 (PMC9218101; doi:10.3389/fcvm.2022.858097)
Supplement: Supplementary file 1 [file Data_Sheet_1.docx]

**Supplemental Material**

**Contents**

**Ⅰ** **Supplemental Tables**

**Table S1:** Baseline clinical characteristics of the study patients according to HAM-A score ≥14.

**Table S2:** Logistic analysis of anxiety for LVH in subgroups.

**Table S3**: Linear regression model of HAM-A score for Tp-Te/QT ratio in subgroups.

**Ⅱ Supplemental Figure**

**Figure S1.** The correlation between LVMI and Tp-Te/QT ratio.

**Table S1: Baseline clinical characteristics of the study patients according to HAM-A score ≥14.**

|  | Total | HAM-A score <14 | HAM-A score ≥14 | *P* value |
| --- | --- | --- | --- | --- |
|  | n=353 | n=278 | n=75 |  |
| Age, year | 59.29±9.87 | 59.25±9.52 | 59.45±11.15 | 0.848 |
| Male, n % | 177(50.1) | 149(53.6) | 28(37.3) | 0.012 |
| BMI, kg/m^2^ | 24.47±3.11 | 24.39±3.04 | 24.78±3.35 | 0.919 |
| Heart rate, bpm | 73.80±10.27 | 73.78±10.17 | 73.85±10.68 | 0.975 |
| SBP, mmHg | 131.95±18.48 | 127.40±14.26 | 148.81±22.30 | <0.001 |
| DBP, mmHg | 79.65±11.59 | 77.56±9.94 | 87.39±13.88 | <0.001 |
| Cigarette smoking, n % | 95(26.9) | 78(28.1) | 17(22.7) | 0.350 |
| Uric acid, umol/L | 314.17±88.10 | 313.68±85.09 | 315.96±99.06 | 0.780 |
| Creatinine, mmol/L | 65.99±15.76 | 66.55±15.74 | 63.92±15.74 | 0.196 |
| TG, mmol/L | 1.69±1.01 | 1.64±0.91 | 1.86±1.32 | 0.377 |
| TC, mmol/L | 4.16±1.07 | 4.15±1.06 | 4.17±1.08 | 0.934 |
| HDL-C, mmol/L | 1.15±0.28 | 1.14±0.27 | 1.16±0.31 | 0.733 |
| LDL-C, mmol/L | 2.33±0.81 | 2.34±0.79 | 2.31±0.88 | 0.598 |
| IVS, mm | 10.07±0.99 | 10.03±0.93 | 10.23±1.20 | 0.179 |
| LVPW, mm | 9.89±0.89 | 9.87±0.80 | 9.95±1.15 | 0.890 |
| LVEDD, mm | 46.20±4.97 | 46.65±4.35 | 44.52±6.54 | 0.050 |
| LVEF, % | 62.92±3.80 | 63.11±3.83 | 62.21±3.61 | 0.078 |
| Prior medication, n (%) |  |  |  |  |
| ACEI/ARB | 114(32.3) | 84(30.2) | 30(40.0) | 0.108 |
| CCB | 99(28.0) | 70(25.2) | 29(38.7) | 0.021 |
| β blockers | 129(36.5) | 96(34.5) | 33(44.0) | 0.131 |
| Diuretics | 18(5.1) | 12(4.3) | 6(8.0) | 0.198 |
| LVMI, g/m^2^ | 88.90±25.68 | 84.36±23.82 | 105.75±25.45 | <0.001 |
| Tp-Te/QT | 0.24±0.04 | 0.24±0.04 | 0.25±0.05 | 0.038 |

Continuous variables are presented as mean±SD; categorical variables are presented as numbers (percentages);

Abbreviations: BMI, body mass index; DBP, diastolic blood pressure; HAM-A Hamilton anxiety scale; HDL-C, high-density lipoprotein cholesterol; IVS, interventricular septum; LAD, left atrial diameter; LDL-C, low-density lipoprotein cholesterol; LVEDD, left ventricular end-diastolic diameter; LVEF, left ventricular ejection fraction; LVH, left ventricular hypertrophy; LVMI, left ventricular mass index; LVPW, left ventricular posterior wall; QT interval, Q wave start to T wave end interval; SBP, systolic blood pressure; TC, total cholesterol; TG, triglyceride; ACEI, angiotensin converting enzyme inhibitor; ARB, angiotensin II receptor blocker; Tp-Te interval, T-wave peak to T-wave end interval.

**Table S2 Logistic analysis of anxiety for LVH in subgroups**

| LVH | Model 1***** | | |  | Model 2† | | |  | Model 3‡ | | |
| --- | --- | --- | --- | --- | --- | --- | --- | --- | --- | --- | --- |
|  | OR (95% CI) | *P* value | *p-t* |  | OR (95% CI) | *P* value | *p-t* |  | OR (95% CI) | *P* value | *p-t* |
| Sex^§^ |  |  | 0.035 |  |  |  | 0.032 |  |  |  | 0.040 |
| Male (n=177) | 5.73(2.43-13.50) | <0.001 |  |  | 5.73(2.42-13.56) | <0.001 |  |  | 5.56(2.07-14.98) | 0.001 |  |
| Female (n=176) | 1.70(0.82-3.53) | 0.152 |  |  | 1.65(0.79-3.43) | 0.185 |  |  | 1.44(0.64-3.26) | 0.377 |  |
| Age^§^ |  |  | 0.380 |  |  |  | 0.296 |  |  |  | 0.275 |
| <60 years (n=179) | 3.91(1.79-8.54) | 0.001 |  |  | 3.97(1.76-8.92) | 0.001 |  |  | 3.91(1.58-9.67) | 0.003 |  |
| ≥60 years (n=174) | 2.13(0.98-4.63) | 0.055 |  |  | 2.04(0.93-4.46) | 0.076 |  |  | 1.53(0.65-3.64) | 0.333 |  |
| BMI^§^ |  |  | 0.551 |  |  |  | 0.539 |  |  |  | 0.221 |
| <24 kg/m^2^ (n=151) | 3.41(1.52-7.63) | 0.003 |  |  | 3.41(1.51-7.67) | 0.003 |  |  | 3.97(1.62-9.73) | 0.003 |  |
| ≥24 kg/m^2^ (n=202) | 2.44(1.15-5.15) | 0.020 |  |  | 2.29(1.06-4.92) | 0.034 |  |  | 1.50(0.63-3.55) | 0.357 |  |
| Smoking^§^ |  |  | 0.173 |  |  |  | 0.155 |  |  |  | 0.333 |
| Yes (n=95) | 5.63(1.83-17.30) | 0.003 |  |  | 5.79(1.84-18.25) | 0.003 |  |  | 4.10(1.04-16.07) | 0.043 |  |
| No (n=258) | 2.30(1.22-4.32) | 0.010 |  |  | 2.23(1.18-4.24) | 0.014 |  |  | 2.03(1.02-4.05) | 0.044 |  |

***** Model 1: unadjusted.

† Model 2: adjusted for age, sex, body mass index, cigarette smoking.

‡ Model 3: adjusted for age, sex, body mass index, cigarette smoking, creatinine, uric acid, total cholesterol, triglyceride, low-density lipoprotein cholesterol, high-density lipoprotein cholesterol, ACEI/ARB, β blockers, and calcium channel blockers.

§ Analyses were adjusted for covariates age, sex, body mass index, cigarette smoking, creatinine, uric acid, total cholesterol, triglyceride, low-density lipoprotein cholesterol, high-density lipoprotein cholesterol, ACEI/ARB, β blockers, and calcium channel blockers when they were not the strata variables.

Abbreviations: ACEI, angiotensin converting enzyme inhibitor; ARB, angiotensin II receptor blocker; BMI, body mass index; CI, confidence interval; LVH, left ventricular hypertrophy; OR, odds ratio; *p-t*, *p* for Interaction.

**Table S3 linear regression model of HAM-A score for Tp-Te/QT ratio in subgroups.**

| Tp-Te/QT | Model 1***** | | |  | Model 2† | | |  | Model 3‡ | | |
| --- | --- | --- | --- | --- | --- | --- | --- | --- | --- | --- | --- |
|  | β (95% CI) | *p* value | *p-t* |  | β (95% CI) | *p* value | *p-t* |  | β (95% CI) # | *p* value | *p-t* |
| Sex^§^ |  |  | 0.014 |  |  |  | 0.017 |  |  |  | 0.045 |
| Male (n=177) | 0.003(0.002-0.004) | <0.001 |  |  | 0.003(0.002-0.004) | <0.001 |  |  | 0.002(0.001-0.003) | <0.001 |  |
| Female (n=176) | 0.001(-0.0001-0.002) | 0.061 |  |  | 0.001(0.0001-0.002) | 0.050 |  |  | 0.001(-0.0002-0.002) | 0.165 |  |
| Age^§^ |  |  | 0.595 |  |  |  | 0.633 |  |  |  | 0.574 |
| <60 years (n=179) | 0.002(0.001-0.003) | 0.001 |  |  | 0.002(0.001-0.003) | <0.001 |  |  | 0.002(0.001-0.003) | 0.002 |  |
| ≥60 years (n=174) | 0.002(0.001-0.002) | 0.001 |  |  | 0.002(0.001-0.003) | <0.001 |  |  | 0.001(0.0004-0.002) | 0.003 |  |
| BMI^§^ |  |  | 0.635 |  |  |  | 0.669 |  |  |  | 0.794 |
| <24 kg/m^2^ (n=151) | 0.002(0.001-0.003) | 0.004 |  |  | 0.002(0.001-0.003) | <0.001 |  |  | 0.001(0.0002-0.002) | 0.021 |  |
| ≥24 kg/m^2^ (n=202) | 0.002(0.001-0.003) | <0.001 |  |  | 0.002(0.001-0.003) | <0.001 |  |  | 0.002(0.001-0.003) | 0.001 |  |
| Smoking^§^ |  |  | 0.367 |  |  |  | 0.440 |  |  |  | 0.575 |
| Yes (n=95) | 0.002(0.001-0.004) | 0.002 |  |  | 0.002(0.001-0.004) | 0.002 |  |  | 0.002(0.0005-0.004) | 0.011 |  |
| No (n=258) | 0.002(0.001-0.0024) | <0.001 |  |  | 0.002(0.001-0.003) | <0.001 |  |  | 0.001(0.0005-0.002) | 0.002 |  |

***** Model 1: unadjusted.

† Model 2: adjusted for age, sex, body mass index, cigarette smoking.

‡ Model 3: adjusted for age, sex, body mass index, cigarette smoking, creatinine, uric acid, total cholesterol, triglyceride, low-density lipoprotein cholesterol, high-density lipoprotein cholesterol, ACEI/ARB, β blockers, and calcium channel blockers.

# Adjusted for Model 3 plus LVH.

§ Analyses were adjusted for covariates age, sex, body mass index, cigarette smoking, creatinine, uric acid, total cholesterol, triglyceride, low-density lipoprotein cholesterol, high-density lipoprotein cholesterol, ACEI/ARB, β blockers, and calcium channel blockers when they were not the strata variables.

Abbreviations: ACEI, angiotensin converting enzyme inhibitor; ARB, angiotensin II receptor blocker; BMI, body mass index; CI, confidence interval; HAM-A Hamilton anxiety scale; OR, odds ratio; *p-t*, *p* for Interaction; QT interval, Q wave start to T wave end interval; Tp-Te interval, T-wave peak to T-wave end interval.


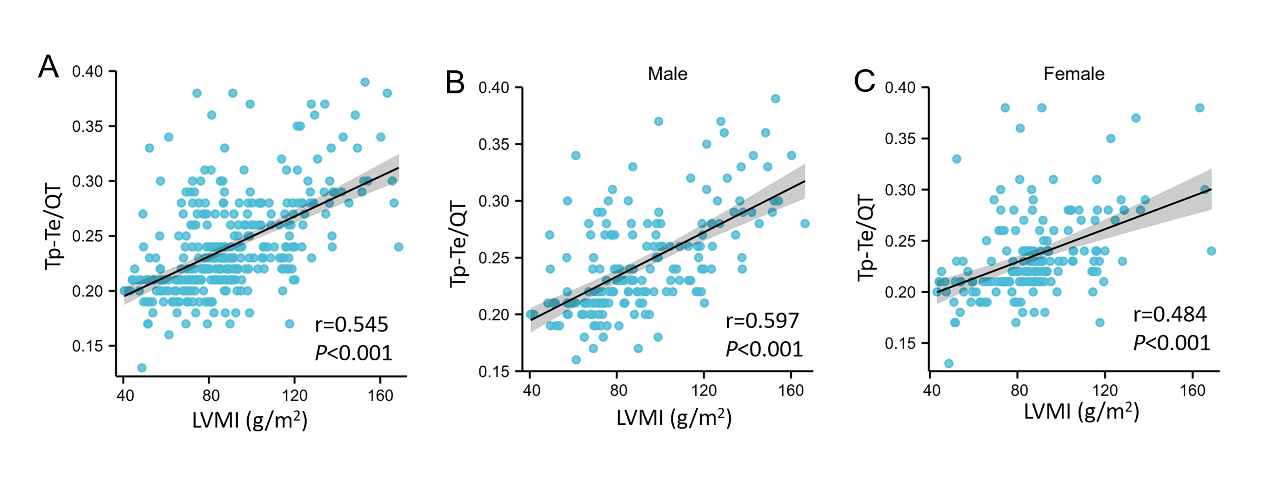


**Figure S1: The correlation between LVMI and Tp-Te/QT ratio.**

**(A)** LVMI was positively correlated with Tp-Te/QT ratio (r=0.545, *P*<0.001). **(B)** and **(C)** After stratification by sex, LVMI remains positively correlated with Tp-Te/QT ratio (male: r=0.597, *P*<0.001; female: r=0.484, *P*<0.001).

Abbreviations: HAM-A Hamilton anxiety scale; LVMI, left ventricular mass index; QT interval, Q wave start to T wave end interval; Tp-Te interval, T-wave peak to T-wave end interval.
